# Gut Health Evaluation Using Gut Microbiome and Intestinal Alkaline Phosphatase Levels in Postweaning Diarrhoea

**DOI:** 10.1155/vmi/4499017

**Published:** 2025-10-09

**Authors:** Pakamas Tansarawut, Prapassorn Boonsoongnern, Attapon Kamlangdee, Yonlayong Woonwong, Alongkot Boonsoongnern

**Affiliations:** ^1^Veterinary Clinical Study Program, Faculty of Veterinary Medicine, Kasetsart University, Kamphaeng Saen Campus, Nakhon Pathom, Thailand; ^2^Department of Anatomy, Faculty of Veterinary Medicine, Kasetsart University, Bangkok, Thailand; ^3^AK Sci and Consulting, Pathum Thani, Thailand; ^4^Department of Farm Resources and Production Medicine, Kasetsart University, Nakhon Pathom, Thailand

**Keywords:** faecal microbiota, gut health, intestinal alkaline phosphatase, nursery pigs, postweaning diarrhoea

## Abstract

Postweaning diarrhoea (PWD) poses a significant threat to the swine industry by causing notable declines in productivity and mortality. Gut health diagnosis in pigs typically involves complex methods such as gut microbiome analysis, which can be costly and can require specialised skills. This study aimed to assess gut health in postweaning piglets by measuring and comparing gut microbiome profiles and levels of intestinal alkaline phosphatase (IALP) in faecal samples from pigs with and without PWD. This study revealed significant differences between the nondiarrhoea and diarrhoea groups of piglets in terms of IALP levels and gut microbiome composition. Nondiarrhoeal piglets had greater IALP levels than did diarrhoeal piglets (*p*=0.003). Additionally, faecal flora richness (observed (*p*=0.0007) and Chao1 (*p*=0.0007)) indices of the faecal microflora in the nondiarrhoeal pigs. At the phylum level, *Firmicutes* and *Bacteroidetes* were predominantly abundant in both groups, while *Firmicutes* (*p*=0.0008) and *Patescibacteria* (*p*=0.0334) showed significantly lower abundances in the nondiarrhoea group and *Bacteroidetes* (*p*=0.0003) exhibited greater abundance. The *Clostridia* class was significantly more abundant in the diarrhoea group than the nondiarrhoea group (*p*=0.0159). The diarrhoea group had a significantly greater relative abundance of the *Clostridiaceae* family than did the nondiarrhoea group (*p*=0.0007). At the genus level, the relative abundance of *Prevotellaceae* NK3B31 was significantly greater in the nondiarrhoea group than the diarrhoea group (*p*=0.0032). Moreover, the relative abundances of some pathogenic bacteria, including *Clostridium* sensu stricto (*Clostridiaceae*) 1, were significantly greater in the diarrhoea group than in the nondiarrhoea group (*p*=0.0007). IALP levels and gut microbiome diversity in faecal samples can be used to assess the gut health of nursery pigs. These results contribute to the understanding and manipulation of postweaning piglet gut health.

## 1. Introduction

Postweaning diarrhoea (PWD) represents one of the most serious threats to the swine industry worldwide as it leads to productivity loss and increased mortality [1]. PWD is a multifactorial disease with a predominantly infectious basis due to gram-negative bacteria such as *Escherichia coli* (especially enterotoxigenic strains, ETEC), *Campylobacter* spp. and *Salmonella* spp., as well as rotavirus [2]. Management practices also play a crucial role, as postweaning changes, such as alterations in the type of food and implementation of social and environmental modifications, can impact the digestive tract. These changes in the digestive tract are followed by improvements in gastrointestinal health. “Gastrointestinal health,” also known as “gut health,” plays a crucial role in the overall well-being of a pig, including its ability to effectively digest food and absorb nutrition. It also aids in the prevention of diseases by maintaining cellular tight junctions, enhancing the epithelial barrier, and promoting the secretion of mucins and immunoglobulins. By preventing disease, these practices ultimately enhance production efficiency. Currently, the main methods for diagnosing gut health in pigs include faecal scores, necropsy findings, pathogenic and non-pathogenic bacterial cultures, histopathology, tight junction analyses, and gut microbiome analyses.

These gastrointestinal microbial communities are commonly referred to as microbiota or microbiomes, and they are critically important for the overall health of pigs [3, 4]. The gut microbiome can improve feed efficiency and growth performance, produce antimicrobial substances, neutralise toxins [5], and improve herd health [3]. The profile of the gut microbiota is an additional indicator of intestinal health and warrants further research [4]. Research on the gut microbiome in pigs is gaining popularity. Various studies have assessed the gut microbiomes in pigs in relation to infection caused by different diseases. Some of these pathogens include African swine fever (ASF) virus [6], *Balantidium coli* (*B. coli*) [7], porcine delta coronavirus (PDCoV) [8], porcine rotavirus (PoRV) [9], *Ascaris suum* [10], porcine epidemic diarrhoea (PED) virus [11], and enterotoxigenic *Escherichia coli* [12], among others. Most reports assess alpha diversity, which indicates species richness based on diversity indices, as well as the composition of the gut microbiome at the phylum, family, and genus levels [2, 6–10]. The gut microbiome may be difficult for farmers to assess due to the high cost and a need for skilled professionals to conduct the tests and perform advanced techniques. Therefore, researchers have explored and identified alternative methods for diagnosing gut health, namely, measuring the levels of intestinal alkaline phosphatase (IALP).

IALP is a brush border enzyme [13] that is produced by intestinal epithelial cells and plays a role in the maintenance of intestinal health [14] and intestinal barrier function through its ability to dephosphorylate lipopolysaccharide (LPS) [15] and prevent and reduce intestinal inflammation [16, 17]. In humans, IALP expression is defective in patients with gastrointestinal disorders such as inflammatory bowel disease [18], necrotising enterocolitis [19], and metabolic syndrome [20]. IALP levels are also used as a biomarker for assessing gut health and as a noninvasive testing method through patient excrement collection [19]. The study of IALP in pigs is limited, particularly the use of IALP as a feed additive, which has been described as a new nutritional therapy [21].

This study aimed to evaluate gut health by measuring the gut microbiome profile and IALP levels in faecal samples from piglets with and without PWD.

## 2. Materials and Methods

### 2.1. Ethical Approval

This research project was approved by the Institutional Animal Care and Use Committee, Faculty of Veterinary Medicine, Kasetsart University (ACKU66-VET-070). Moreover, the animal procedures followed ethical guidelines and were approved by the Kasetsart University Institutional Biosafety Committee (IBC, Protocol #IBC-66-V14).

### 2.2. Study Period and Location

The study was conducted in July 2022 in an evaporative house system at the farm demonstration unit, Faculty of Veterinary Medicine, Kasetsart University, Nakhon Pathom Province, Thailand.

### 2.3. Experimental Animals

Twenty Landrace × Large White × Duroc crossbred weaned piglets were purchased at 21 days of age (male and female nursery pigs, with an equal number in each group, had an average body weight of 6.0 kg) from a commercial farm at Phetchabun Province, Thailand. These pigs originated from a farm that was free from infection with three common enteric viruses (porcine transmissible gastroenteritis virus [TGEV], PEDV, and PoRV). All pigs were required to test negative for these three viruses using PCR and ELISA. The pigs were kept in an evaporative housing system. The pen size for housing the pigs was 1.0 × 1.0 m per pen, each containing a single pig, providing a stocking density of 1 m^2^ per pig. The pigs were provided commercial feed and water ad libitum. The experimental diet provided to the pigs contained 3450.0 kcal/kg of ME, along with 22.0% of crude protein and 6.3% of fat from a CARGILL SIAM LIMITED.

**Figure 1:**
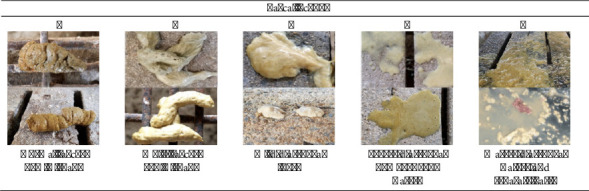
Faecal scores of the piglets. A score of 1-2 indicated normal faeces and an alert pig that appeared to be eating, while a score of 3–5 indicated diarrhoeal faeces.

Pigs were not challenged with any pathogens. The pigs were divided into a nondiarrhoea group (*n* = 10) and a diarrhoea group (*n* = 10) based on the characteristics of their faeces (Figure 1). Fresh faecal samples were collected every morning between 8:30–9:00 a.m. for 2 weeks. Laboratory analyses were conducted within one week after the completion of sample collection from all 20 samples from both the nondiarrhoea faecal group and diarrhoea faecal group before feeding and were scored from 1 to 5 (1 = normal faeces; 2 = moist faeces; 3 = mild diarrhoea; 4 = severe diarrhoea; and 5 = watery diarrhoea).

### 2.4. Sampling

**Figure 2:**
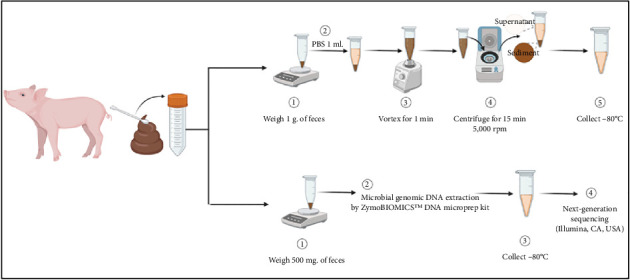
The process of collecting swine faecal samples for IALP ELISA quantification and subsequent processing for next-generation sequencing analysis.

The procedure for swine faecal sample collection involved gathering faecal matter from each pig. Faecal samples were collected directly from the anus of the pigs by allowing spontaneous defecation, followed by collection using a sterile plastic spoon and transfer into a sterile tube for a faecal mass of 3 g/pig. The tubes were then placed in ice-filled containers for transportation to the laboratory. Upon reaching the laboratory, the faecal samples were divided into two portions (Figure 2). The initial portion, weighing 1 g faeces/pig, was deposited into 1.5 mL sterile tubes containing 1 mL of PBS solution. After vortexing for 1 min, the tubes were centrifuged for 15 min at 5000 rpm, and the supernatant was stored at −80°C for subsequent IALP ELISA analysis following the manufacturer's instructions (MyBioSource®, CA, USA). The second portion, weighing 500 mg faeces/pig, was similarly deposited into sterile 1.5 mL tubes and stored at −80°C until genomic DNA extraction.

### 2.5. Faecal DNA Extraction

Microbial genomic DNA was extracted from the swine faecal samples using a ZymoBIOMICS^TM^ DNA Microprep Kit (Zymo Research, CA, USA) following the manufacturer's instructions. The final concentration and purity of each DNA sample were determined using a NanoDrop 2000 spectrophotometer (Thermo Scientific, DE, USA), and then, the samples were stored at −20°C until next-generation sequencing (Illumina, CA, USA).

### 2.6. Microbiota Diversity Analysis

16S (V3-V4) rRNA was amplified from a DNA sample and then sequenced using an Illumina MiSeq platform (Illumina, CA, USA). For the taxonomic assignment of sequence reads, the output data from an Illumina iSeq sequencing system were analysed using the Silva 138.1 database for taxonomic classification, and DADA2 v1.20.0 is a pipeline for processing amplicon sequence variants (ASVs). The ASVs were clustered more than 97% similarity threshold using Phyloseq v1.38.0. For microbial alpha diversity analysis, the Observed, Chao1, Shannon, and Simpson indices were calculated to determine the community richness and diversity of the faecal microbiota using Phyloseq v1.38.0.

### 2.7. IALP ELISA

According to the manufacturer's instructions, the faecal samples were analysed in duplicate by competitive enzyme-linked immunosorbent assays (ELISA). The concentration of IALP was determined using an IALP ELISA test kit (lot number 20230731C) from MyBioSource®, CA, USA. Briefly, a competitive ELISA method with a detection limit of 0.1 ng/mL was employed. Initially, 100 μL of the standard, sample, or PBS was added to each well and was designated as the positive control, sample, or negative control, respectively. Then, 10 μL of balance solution was added to the sample wells. Subsequently, 50 μL of conjugate was added to all wells, followed by incubation at 37°C for 1 h. The plate was washed with 1X wash solution 5 times and blotted dry with tissue paper. Next, 50 μL of substrate A and 50 μL of substrate B were added to all wells and incubated at 37°C for 15–20 min. Afterwards, 50 μL of stop solution was added to each well. The optical density (O.D.) was read immediately at 450 nm using a microplate reader. The standard curve was generated from the O.D. values of six different concentrations of the standard, which were then used to calculate the IALP concentrations (ng/mL) in the samples.

### 2.8. Statistical Analysis

The relative abundance of different taxa, the alpha diversity, and IALP levels in diarrhoea faeces and nondiarrhoea faeces were compared using *t* tests and Mann–Whitney *U* tests in R statistical software (v4.3.1; R Core Team (2023). R: A language and environment for statistical computing (R Foundation for Statistical Computing, Vienna, Austria, URL https://www.R-project.org/). Normally distributed data were analysed using a *t* test. The Mann–Whitney *U* test was used for nonnormally distributed data. Figures were created using the ggpot2, scales, and tidyr package. All the data are presented as the means ± standard deviations (SD). Differences between two groups were considered statistically significant at *p* ≤ 0.05 (^∗^*p* < 0.05, ^∗∗^*p* < 0.01, ^∗∗∗^*p* < 0.001).

## 3. Results

### 3.1. Comparison of IALP Levels Between the Nondiarrhoea and Diarrhoea Groups

**Figure 3:**
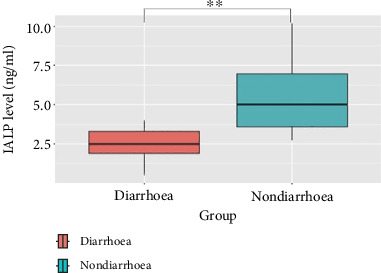
The differences in IALP levels of postweaning piglets. Boxplot of the IALP levels between the two groups showing that the nondiarrhoea group had a significantly greater IALP concentration than did the diarrhoea group (*p*=0.003).

In the nondiarrhoea group of piglets (*n* = 10), the IALP concentrations are 5.508 ± 2.372 ng/mL (range = 2.732–10.137 ng/mL) in terms of the lowest to the highest values and the mean ± SD, respectively. In contrast, the diarrhoea group of piglets (*n* = 10) had 2.484 ± 1.079 ng/mL (range = 0.508–3.989 ng/mL), respectively. These results indicate that the nondiarrhoea group had a significantly greater IALP concentration than did the diarrhoea group (*p*=0.003) (Figure 3).

### 3.2. Comparison of Gut Microbiota Diversity Between the Nondiarrhoea and Diarrhoea Groups

The difference in the diversity of microorganisms between the two groups was determined using a *t* test. There were no significant differences in the diversity indices, such as the Shannon and Simpson diversity indices. Conversely, the richness indices, including the Observed and Chao1 indices, indicated that the faecal flora richness in the nondiarrhoea group was significantly greater than that in the diarrhoea group (*p* < 0.05) (Table 1).

**Figure 4:**
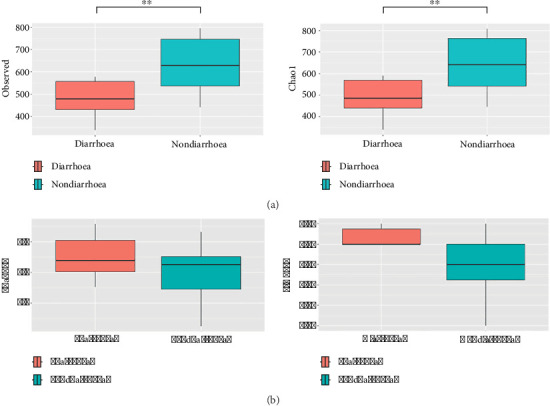
The differences in the gut microbial alpha diversity between the nondiarrhoea group and the diarrhoea group were determined by the (a) observed and Chao1 indices and (b) Shannon and Simpson indices. A statistically significant increase in richness diversity was detected in the nondiarrhoea group (^∗^*p* < 0.05, ^∗∗^*p* < 0.01, ^∗∗∗^*p* < 0.001).

The alpha diversity of the gut microbiome differed between the nondiarrhoea group and the diarrhoea group. The observed index was significantly greater in the nondiarrhoea group (632.80 ± 128.59) than in the diarrhoea group (406.40 ± 83.37) (*p*=0.0007). Similarly, the Chao1 index was significantly greater in the nondiarrhoea group (643.41 ± 133.12) than in the diarrhoea group (410.98 ± 84.20) (*p*=0.0007) (Figure 4(a)). When the Shannon and Simpson diversity indices were compared, the differences between the two groups were not significant (Figure 4(b)).

### 3.3. Comparison of Bacterial Microbiota Compositions Between the Nondiarrhoea and Diarrhoea Groups

**Figure 5:**
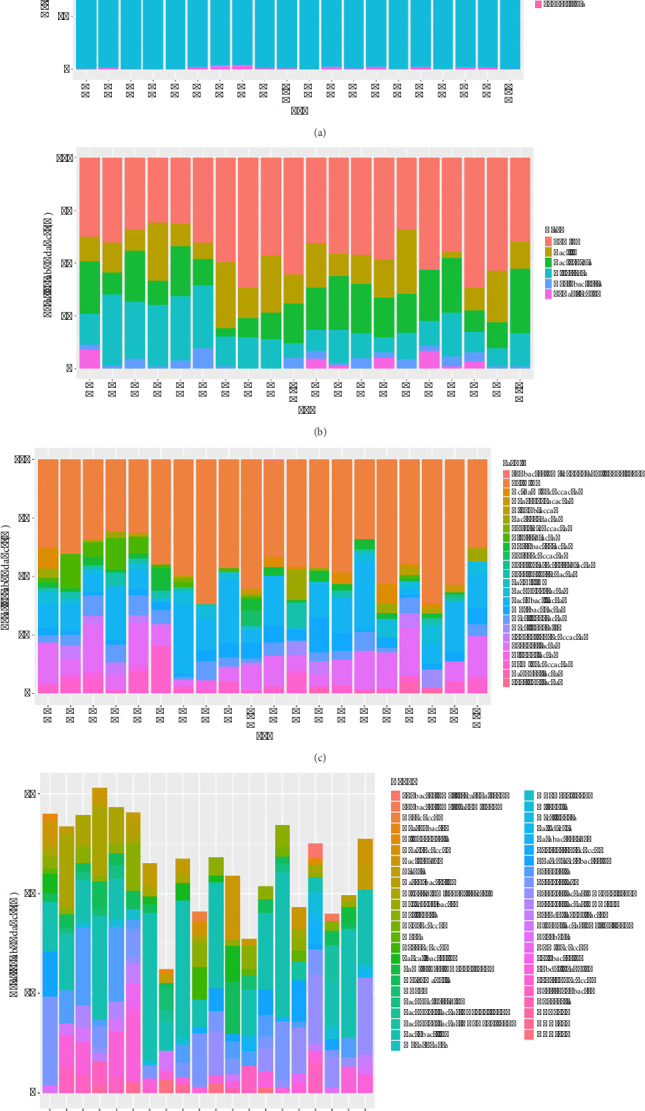
The relative abundance at the phylum (a), class (b), family (c), and genus (d) levels of the faecal microbiome in postweaning piglets of the nondiarrhoea group (N1–N10) and diarrhoea group (D1–D10).

A total of 14 phyla were identified. *Elusimicrobia*, *Fusobacteria*, and *Fibrobacteres* were found exclusively in the nondiarrhoea group. The phyla *Firmicutes* and *Bacteroidetes* were predominant in both groups, together accounting for more than 94% of the relative abundance. The relative abundances of *Firmicutes* (*p*=0.0008) and *Patescibacteria* (*p*=0.0334) were significantly lower in the nondiarrhoea group than in the diarrhoea group. Conversely, the phylum *Bacteroidetes* (*p*=0.0003) exhibited a significantly greater relative abundance in the nondiarrhoea group than in the diarrhoea group (Figure 5(a)).

At the class level, there were a total of 5 classes with a relative abundance greater than 1%. *Elusimicrobia*, *Fusobacteria*, and *Fibrobacteres* were found exclusively in the nondiarrhoea group. Conversely, *Chlamydiae* and *Verrucomicrobiae* were found exclusively in the diarrhoea group. There was a noticeable difference between the two groups at the class level. The relative abundance of the *Clostridia* class in the diarrhoea group was significantly greater than that in the nondiarrhoea group (*p*=0.0159) (Figure 5(b)).

At the family level, 8 families were found exclusively in the nondiarrhoea group, including *Barnesiellaceae*, *Bifidobacteriaceae*, *Coriobacteriaceae Incertae Sedis*, *Elusimicrobia*, *Fibrobacteres*, *Fusobacteria*, *Propionibacteriaceae*, and *Pseudomonadaceae*. However, 12 families (*Actinomycetaceae*, *Alcaligenaceae*, *Chlamydiaceae*, *Corynebacteriaceae*, *Family XI*, *Morganellaceae*, *Paracaedibacteraceae*, *Pasteurellaceae*, *Sphingobacteriaceae*, *Staphylococcaceae*, and *Streptomycetaceae*) were found only in the diarrhoea group. There were a total of 23 families with a relative abundance greater than 1%. Moreover, the diarrhoea group had a significantly greater relative abundance of *Clostridiaceae* than did the nondiarrhoea group (*p*=0.0007) (Figure 5(c)).

At the genus level, the relative abundances (relative abundance > 1%) of 47 genera showed that *Lactobacillus* was the predominant genus in both groups. In particular, the relative abundance of the *Prevotellaceae* NK3B31 group was significantly greater in the nondiarrhoea group (*p*=0.0032). Moreover, the relative abundances of some pathogenic bacteria, including *Clostridium* sensu stricto 1 (*Clostridiaceae*), were significantly greater in the diarrhoea group (*p*=0.0007) (Figure 5(d)).

## 4. Discussion

After weaning, piglets are easily exposed to various stress factors, such as physiological, environmental, social, and nutritional changes [22]. Some weaned pigs showed clinical signs of diarrhoea in this study, which aligns with prior research findings indicating that once piglets are weaned, their feed intake decreases, susceptibility to diarrhoea incidence, stunted growth, reduced disease resistance, and increased risk for later inflammatory and metabolic diseases. These negative effects not only impact the first week after weaning but also have long-term negative effects on swine productivity [3, 23, 24]. To enhance researchers' understanding of gut health postweaning, it is essential to elucidate the intricacies of the digestive system. Profound comprehensions of both typical and atypical conditions, such as diarrhoea, that arise within the digestive environment are necessary, including assessment of the gut microbiome and IALP levels. These findings will pave the way for identifying effective solutions and ultimately enhancing gut health to improve swine farm production.

### 4.1. IALP Levels in Postweaning Pigs With Diarrhoea

The low levels of IALP in pigs with PWD are similar to findings in humans, which suggests that individuals with gastrointestinal illnesses have lower levels of IALP than healthy individuals [13, 18, 25]. IALP is produced by enterocytes and is typically secreted in greater amounts when the host consumes a high-fibre diet. This soluble fibre is then utilised by bacteria to produce short-chain fatty acids (SCFAs) [26, 27], leading to increased IALP secretion in the gastrointestinal tract [15]. In cases of inflammation, IALP can bind to LPS lipid A, preventing the entry of LPS toxin into enterocytes and thus reducing inflammation in the gastrointestinal tract [13, 15]. This pattern of low IALP in piglets with PWD aligns with the idea that healthy piglets have high levels of IALP, which helps prevent gastrointestinal inflammation and subsequently prevents diarrhoea.

Conversely, piglets with low IALP experience gastrointestinal inflammation [15, 28]. Mechanistically, an imbalance in the absorption and secretion of intestinal fluids and electrolytes stimulates this secretion. This results in diarrhoea when the content of Cl^−^ secretion-driven fluid exceeds the absorptive capacity of the intestines, thereby indicating the presence of diarrhoea [16, 29]. Therefore, our findings are consistent with previous studies, as we observed that pigs with diarrhoea exhibited lower levels of IALP.

### 4.2. Gut Microbiota Diversity in Piglets With PWD

We observed high richness indices in the nondiarrhoea group. This finding is consistent with a report by He and colleagues in 2020, which stated that under normal intestinal conditions, a diverse microbiome benefits swine health. In contrast, it has been reported that piglets with gastrointestinal illnesses such as *B. coli* infection have low richness indices [7]. Moreover, in 2023, Ko and colleagues reported that the ASF virus can change the gut microbiome during the disease incubation period. The richness of the bacterial community significantly decreases in the primary phase of ASFV infection [6].

All previous reports have agreed that piglets with lower richness indices tend to be more susceptible to diarrhoea than piglets with higher richness indices. Due to the microbial diversity that piglets inherit from sows, more diverse strains are present at younger ages [3, 10]. This renders them well prepared to handle various changes, including changes in diet, social interactions, and diseases. Having a more diverse bacterial population allows the piglets to digest a wider variety of feeds or maintain bacteria that can utilise a wider variety of nutrients, leaving fewer leftover nutrients for pathogenic bacteria or certain groups of bacteria to overgrow, which can then lead to diarrhoea [30, 31]. Therefore, these findings support the idea that weaned pigs from sows with more diverse bacterial strains have lower faecal flora richness of diarrhoea.

### 4.3. Gut Microbiota Composition in Piglets With PWD

The gut microbiome composition in piglets with PWD was dominated by the phyla *Firmicutes* and *Bacteroidetes*, which is consistent with findings from other studies that have examined the gut microbiome composition [2, 7, 10]. Changes in the gut microbiome composition depend on multiple factors, including diet, breed, environment, and age/growth stage [10, 32]. We found that the relative abundance of *Bacteroidetes* was greater in nondiarrhoeal piglets than in piglets with PWD, which is consistent with the results of previous studies on the swine gut microbiome comparing healthy piglets with piglets infected with PED virus [11] and enterotoxigenic *E. coli* [12] *Bacteroidetes* are bacteria that prefer to metabolise polysaccharides and oligosaccharides, providing nutrients and vitamins to the host and other intestinal microbial residents [33]. The postweaning diet in this study consisted mainly of broken rice and soybean. These raw materials are a source of food for bacteria in the *Bacteroidetes* phylum. Piglets with good digestive health, high feed intake, and good digestion have many polysaccharides and oligosaccharides. This finding suggests that piglets with healthy guts will have increased abundances of *Bacteroidetes*. Conversely, weaned piglets that are stressed by various factors eat less, resulting in fewer food sources for *Bacteroidetes* and a lower relative abundance of *Bacteroidetes* in weaned piglets with diarrhoea or poor gut health.

The abundance of *Chlamydiae* was consistent with that at the family level, and the *Chlamydiaceae* family was found only in piglets with diarrhoea. *Chlamydiaceae* are obligate intracellular, gram-negative bacteria that cause a broad range of diseases in animals and humans. *Chlamydia suis*, *Chlamydia abortus*, *Chlamydia pecorum*, and *Chlamydia psittaci* have been isolated from swine. Chlamydial infections in swine are associated with respiratory disease, diarrhoea, conjunctivitis, and other pathologies [34–36]. In the group of pigs exhibiting diarrhoea, *Chlamydiaceae* was detected, which aligns with previous studies mentioned earlier. Therefore, close monitoring of pigs showing symptoms of diarrhoea is recommended, as the presence of *Chlamydiaceae* may indicate an increased risk of illness.

Moreover, the abundance of *Clostridiaceae* was observed to increase significantly in diarrhoeal piglets, which is consistent with the results of a study of PED-related diarrhoea, which also revealed that Clostridia and PED coinfection is more strongly associated with diarrhoea severity in weaned piglets [37]. *Clostridia* occupy the gastrointestinal tract from birth until the sixth hour and shift in relative abundance to *Lactobacillus* in 1–3 days [38, 39]. However, in piglets that still have high *Clostridia* levels during the postweaning period, this may be due to a high intake of fermented protein or indigestible proteins [40]. These foods are a favourite food source for *Clostridia*, resulting in high levels of *Clostridia* during the postweaning period. Many studies have reported that *Clostridia* is an opportunistic bacterium and causes important gastrointestinal diseases in both swine and humans [41]. The results of this study indicate that pigs with diarrhoea combined with high abundances of *Chlamydiaceae* and *Clostridiaceae* may experience adverse health effects, and such infections could lead to complications and negatively impact future production efficiency.

Previous studies have found that the abundance of the *Prevotellaceae* NK3B31 group is associated with SCFAs and polyamine levels [42] and is highly correlated with viral load and weight gain after porcine reproductive and respiratory syndrome (PRRS) viral infection [43]. Wang and colleagues suggested that the gut microbiota, particularly abundance of the *Prevotellaceae* NK3B31 group, significantly positively impacts immune function and growth performance, indicating a potential link to viremia, clinical symptoms, and disease resistance [43]. The presence of *Prevotellaceae* NK3B31 in the gastrointestinal tract of nursery pigs aids in providing energy for growth and maintenance of a healthy gut. Abundance of this group also promotes growth by reducing inflammation, decreasing intestinal permeability, degrading proteins and carbohydrates, producing intramuscular fats, and storing hepatic glycogen, thereby contributing to weight gain in pigs [44–46]. Thus, the results of many studies, along with our results, have revealed that abundance of the *Prevotellaceae* NK3B31 group is positively associated with piglet health and performance.

### 4.4. Suggested Links Between the Gut Microbiome and IALP Levels

**Figure 6:**
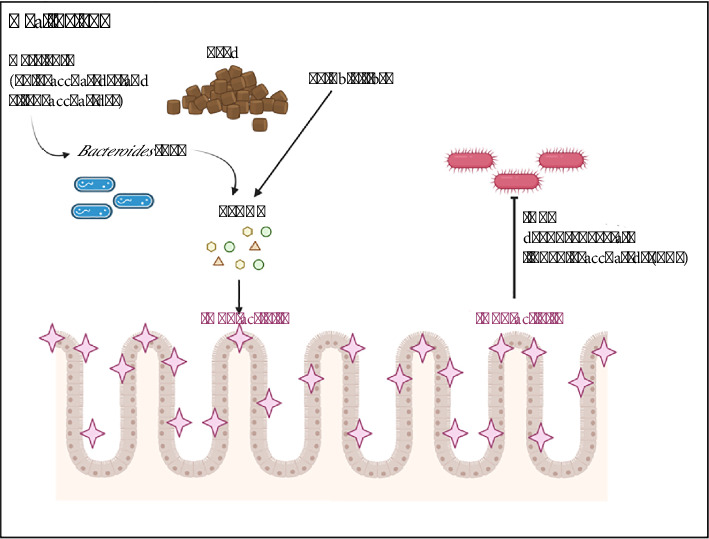
Impact of feed on intestinal alkaline phosphatase (IALP) activity. Polysaccharides, oligosaccharides, and soluble fibre in swine feed provide substrates for the gut microbiome, leading to fermentation processes that produce SCFAs. This fermentation enhances IALP activity. Subsequently, IALP dephosphorylates LPS from gram-negative bacteria, thereby preventing the induction of inflammation in the gastrointestinal tract.

In swine with an intact gastrointestinal system, dietary polysaccharides and oligosaccharides serve as nutrient sources for the *Bacteroides* group, which are the primary producers of SCFAs [33, 47, 48]. SCFAs stimulate enterocytes to secrete IALP, which subsequently binds to LPS lipid A, thereby preventing gram-negative bacteria from binding to receptors and triggering inflammation [15] (Figure 6). Conversely, in swine experiencing gastrointestinal distress and dysbiosis, SCFAs production is reduced, leading to decreased IALP secretion. This allows gram-negative bacteria producing LPS toxins to bind to receptors on enterocytes, thereby initiating an inflammatory response and resulting in diarrhoeal symptoms in swine.

## 5. Conclusions

In this study, we evaluated the gut health of pigs with and without PWD. The evaluation of gut health using the gut microbiome was consistent with that using the IALP levels from faecal samples. PWD and nondiarrhoea pigs had different faecal flora richness, composition, and IALP levels. In the nondiarrhoeal piglets, the IALP levels were greater, the faecal flora richness indices were greater (Observed and Chao1 indices), and the gut microbiome composition showed a higher abundance of the *Bacteroides* phylum and *Prevotellaceae* NK3B31 group but a lower abundance of the *Chlamydiaceae* and *Clostridiaceae* groups. Our findings provide a better understanding of the gut health of postweaning piglets. In conclusion, our findings suggest that faecal IALP levels and microbiome indices can serve as diagnostic tools for evaluating gut health in nursery pigs.

## Figures and Tables

**Table 1:** Comparison of gut microbiota diversity between the nondiarrhoea and diarrhoea groups.

Diversity index		Nondiarrhoea group (*n* = 10)	Diarrhoea group (*n* = 10)	*p* value
Richness indices	Observed	632.80 ± 128.59	406.40 ± 83.37	0.0007
	Chao1	643.41 ± 133.12	410.98 ± 84.20	0.0007

Diversity indices	Shannon	4.62 ± 0.36	4.45 ± 0.28	0.3187
	Simpson	0.97 ± 0.01	0.97 ± 0.01	1.0000

## Data Availability

The data supporting this study are contained within the article and its Supporting Information.

## Nomenclature

ASF: African swine fever
ASVs: Amplicon sequence variants
*B. coli*: *Balantidium coli*
ELISA: Enzyme-linked immunosorbent assays
ETEC: *Escherichia coli* enterotoxigenic strains
D: Diarrhoea groups of piglets
IALP: Intestinal alkaline phosphatase
LPS: Lipopolysaccharide
N: Nondiarrhoea groups of piglets
O.D.: Optical density
PDCoV: Porcine delta coronavirus
PED: Porcine epidemic diarrhoea
PoRV: Porcine rotavirus
PRRS: Porcine reproductive and respiratory syndrome
PWD: Postweaning diarrhoea
SCFAs: Short-chain fatty acids
SD: Standard deviation
TGEV: Porcine transmissible gastroenteritis virus

## Supporting Information

Supporting Information 1 Supporting file 1: Metadata for all samples included in this study.

Supporting Information 2 Supporting file 2: Alpha diversity indices for all samples included in this study.

Supporting Information 3 Supporting file 3: 16S rRNA sequencing reads for diarrhoea group (D1-D10).

Supporting Information 4 Supporting file 4: 16S rRNA sequencing reads for nondiarrhoea group (N1-N10).

Supporting Information 5 Supporting file 5: Intestinal alkaline phosphatase levels for all samples included in this study.
